# Gangrene of the Colon Ascendens, Colon Transversum, and Lienal Flexure in a Massive Strangulated Umbilical Hernia

**DOI:** 10.7759/cureus.30022

**Published:** 2022-10-07

**Authors:** Stanko Baco, Milos Mitric

**Affiliations:** 1 Surgery, Public Health Institution Hospital “Dr Mladen Stojanović”, Prijedor, BIH; 2 General Surgery, Public Health Institution Hospital “Dr Mladen Stojanović”, Prijedor, BIH

**Keywords:** reconstruction of digestive continuity, gangrenous colon, subtotal colectomy, giant hernia, umbilical hernia, strangulation, incarceration

## Abstract

This case describes an 80-year-old obese woman who presented with a giant, incarcerated umbilical hernia. The hernia was present for over 15 years, continuously increasing in size. The only symptom was the pain which lasted about two hours before arrival at the hospital. In an emergency laparotomy, gangrenous colon ascendens, transversum, and lienal flexure have been found. A subtotal colectomy with the creation of terminal ileostomy without hernia repair has been done. The recovery was uneventful, and the patient was discharged on the fifth postoperative day. After one month, the reconstruction of the digestive continuity with an L-L ileo-descendo anastomosis followed. The patient decided against the hernia repair.

## Introduction

A hernia is a protrusion of an organ or a part through connective tissue or through a wall of the cavity in which it is normally enclosed. The umbilical cord, consisting of two arteries and a vein, connects the fetus and placenta and is responsible for the nourishment and oxygen supply from the placenta to the fetus and the removal of waste products [1]. After birth, it is clamped and falls off naturally after a few days. Suppose the abdominal wall does not close solidly, and the umbilical hernia results. European Hernia Society's classification for abdominal wall hernias defines the umbilical hernia as a hernia located from 3 cm above to 3 cm below the umbilicus [2]. The incidence of umbilical hernias in the general adult population is 2%. The female-to-male ratio is 3:1. Nearly 90% of umbilical hernias in adults are acquired with only 10% report having had a hernia in childhood [2-3]. It is the second most common type of hernia in an adult following an inguinal hernia. It accounts for 6%-14% of all abdominal wall hernias in adults [2]. They occur more often in females, multipara, premature infants, and cases of increased intra-abdominal pressure (as in pregnancy, patients with ascites, cirrhosis, and abdominal malignancies) [3]. Umbilical hernias are mostly small, asymptomatic, and contain only omentum [2]. Hernias are classified as reducible when the hernia contents can be placed intra-abdominally through the layers of the abdominal wall. An incarcerated hernia is a hernia in which the content has become irreducible due to a narrow opening in the abdominal wall or due to adhesions between the content and the hernia sac. A strangulated hernia occurs when the hernia contents are ischemic due to a compromised blood supply [4]. The risk of strangulation is estimated at up to 17% and up to three times higher than in femoral hernias [5]. Considering the lack of medical literature reporting umbilical hernia containing colon ascendens, transversum, and descendens we decided to present this case. We think it is important for the readers to see that a big hernial neck and sack, can also cause incarceration and even strangulation, which in older people present with very few symptoms and subtle clinical findings until it becomes very serious [6]. There is a clear message that gangrene of the colon can evolve almost without any symptoms.

## Case presentation

**Figure 1:**
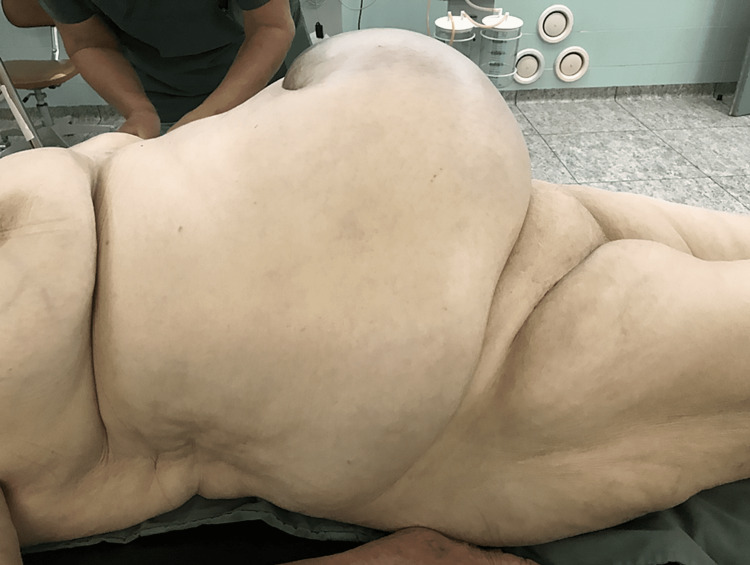
Incarcerated umbilical hernia (right-side view)

**Figure 2:**
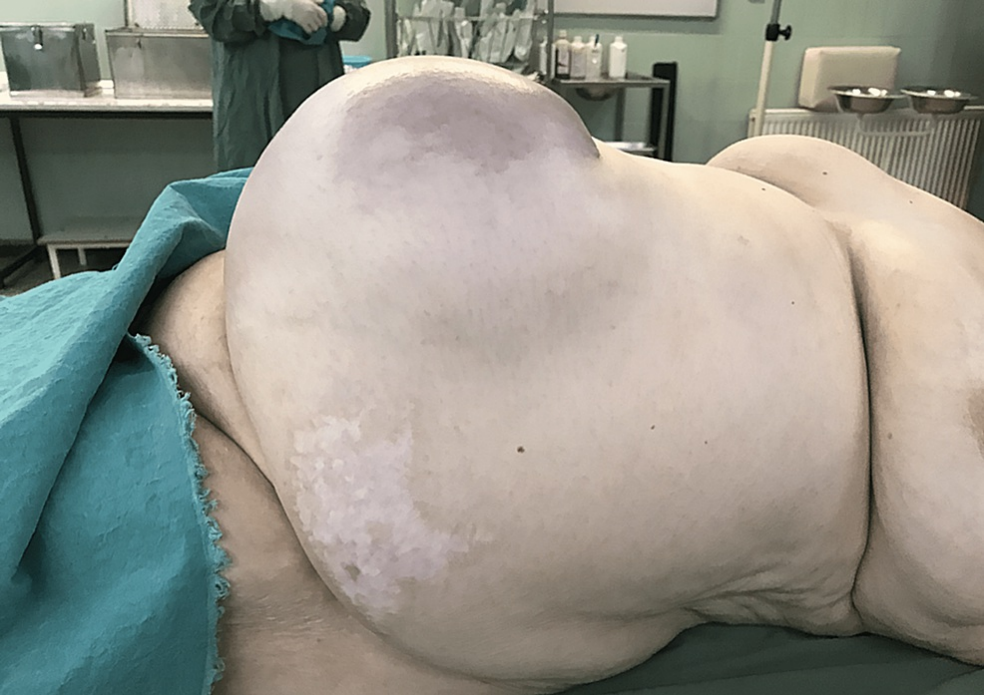
Incarcerated umbilical hernia (left-side view)

**Figure 3:**
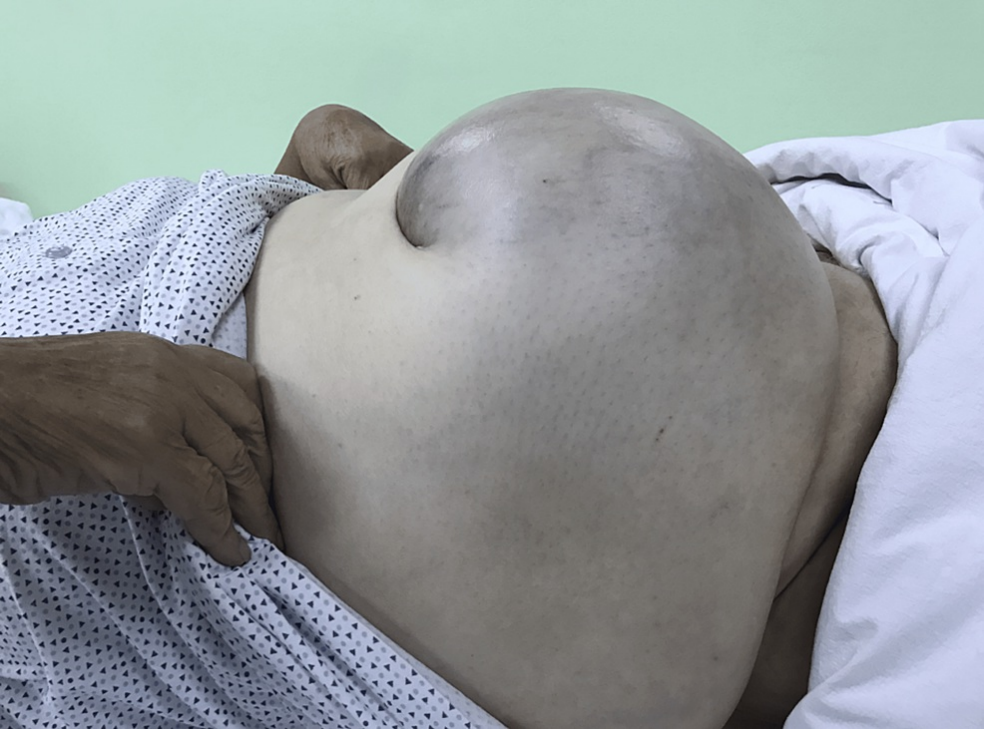
Bluish-colored skin (right-side view)

**Figure 4:**
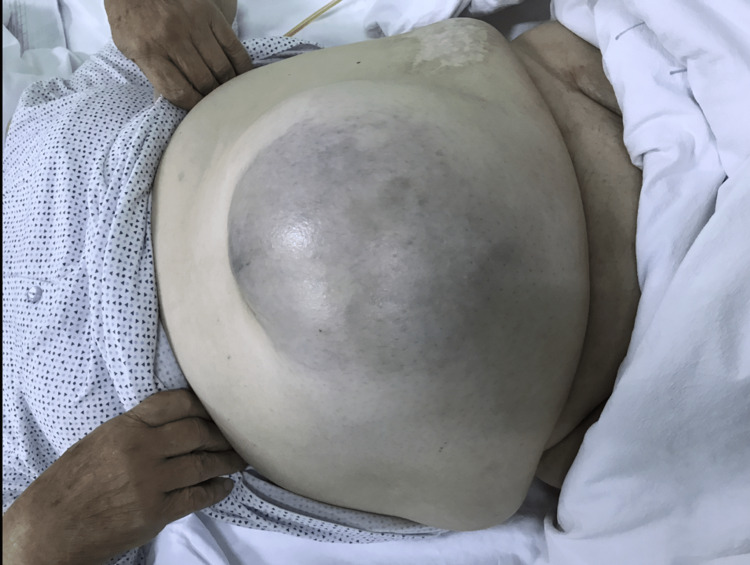
Bluish-colored skin over the incarcerated umbilical hernia (anterior view)

An 80-year-old, obese woman presented in the early morning, to the emergency department with a giant incarcerated umbilical hernia. She complained about abdominal pain, which lasted about two hours before the presentation. The hernia was present for over 15 years, continuously increasing in size; on several occasions reduced by the patient by lying down and applying pressure on the hernia. On examination, there have not been any signs of acute abdomen (guarding or tenderness). An 18 x 18 cm irreducible swelling was present at the umbilicus and infraumbilical. The skin was bluish-colored (Figures 1-4). The patient denied all other symptoms such as nausea, vomiting, or fever, and had had stool three days before. She was obese with a body mass index (BMI) of 32.7 kg/m^2^, had never been operated on, and has regularly taken antihypertensives (Amlopin) and occasionally diuretics and analgesics (NSAID).

**Figure 5:**
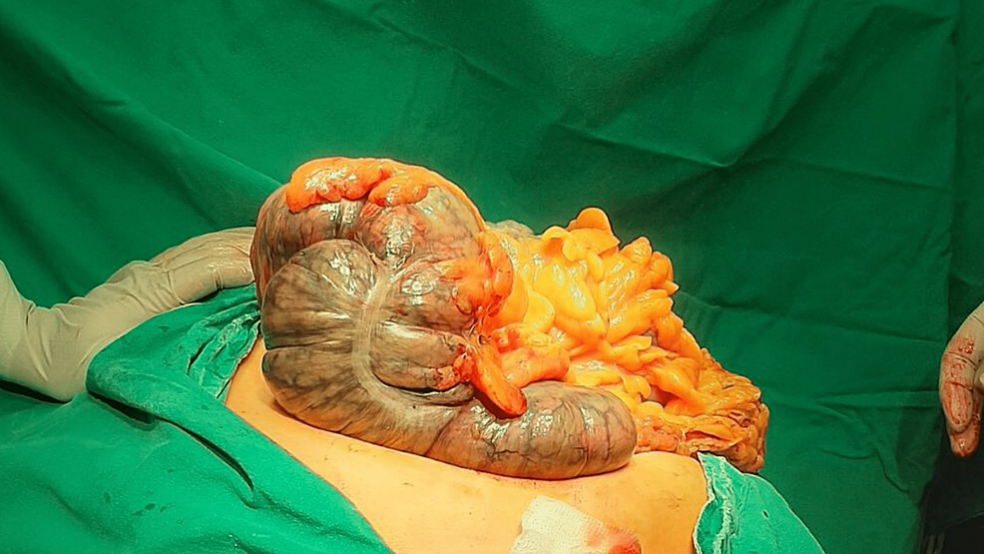
Intraoperative photography of gangrenous colon

The presentation with the incarceration has led us to the decision to operate urgently and not to lose time on an additional investigation (abdomen CT). Intraoperatively we found the ascendant and transverse colon and lineal flexure in the hernia sac, gangrenous but luckily not perforated (Figure 5). The hernia sac was also gangrenous.

**Figure 6:**
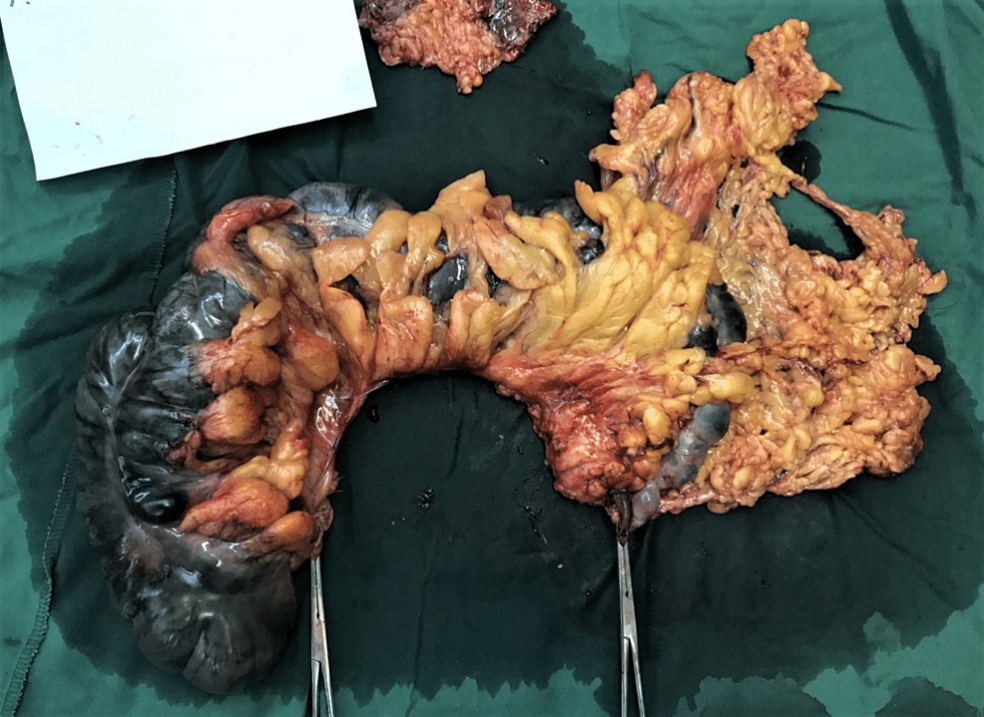
Photography of the specimen

**Figure 7:**
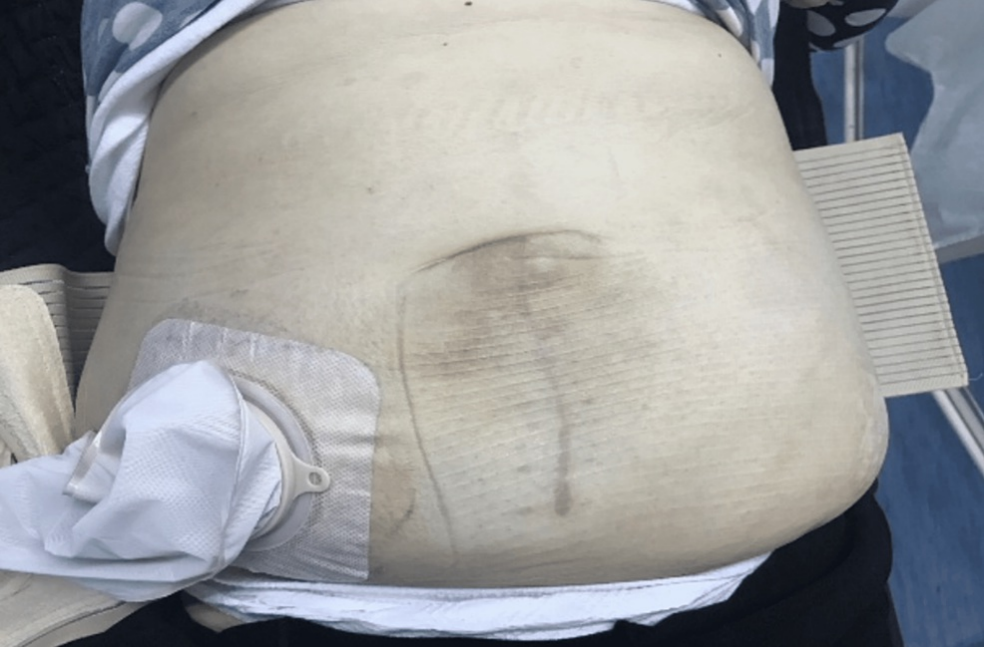
Postoperatively temporary terminal ileostomy and the healed wound

In that environment, we decided against anastomosis creation [7]. We followed the dogma, which not everybody agrees with, of not placing a mesh in a contaminated field. A subtotal colectomy (Figure 6) with the terminal ileostomy creation followed (Figure 7). We have even enlarged the fascial defect (the hernial neck) to reduce the probability of repeated incarceration. Only the skin was sutured. The patient was discharged on the fifth postoperative day without complications.

**Figure 8:**
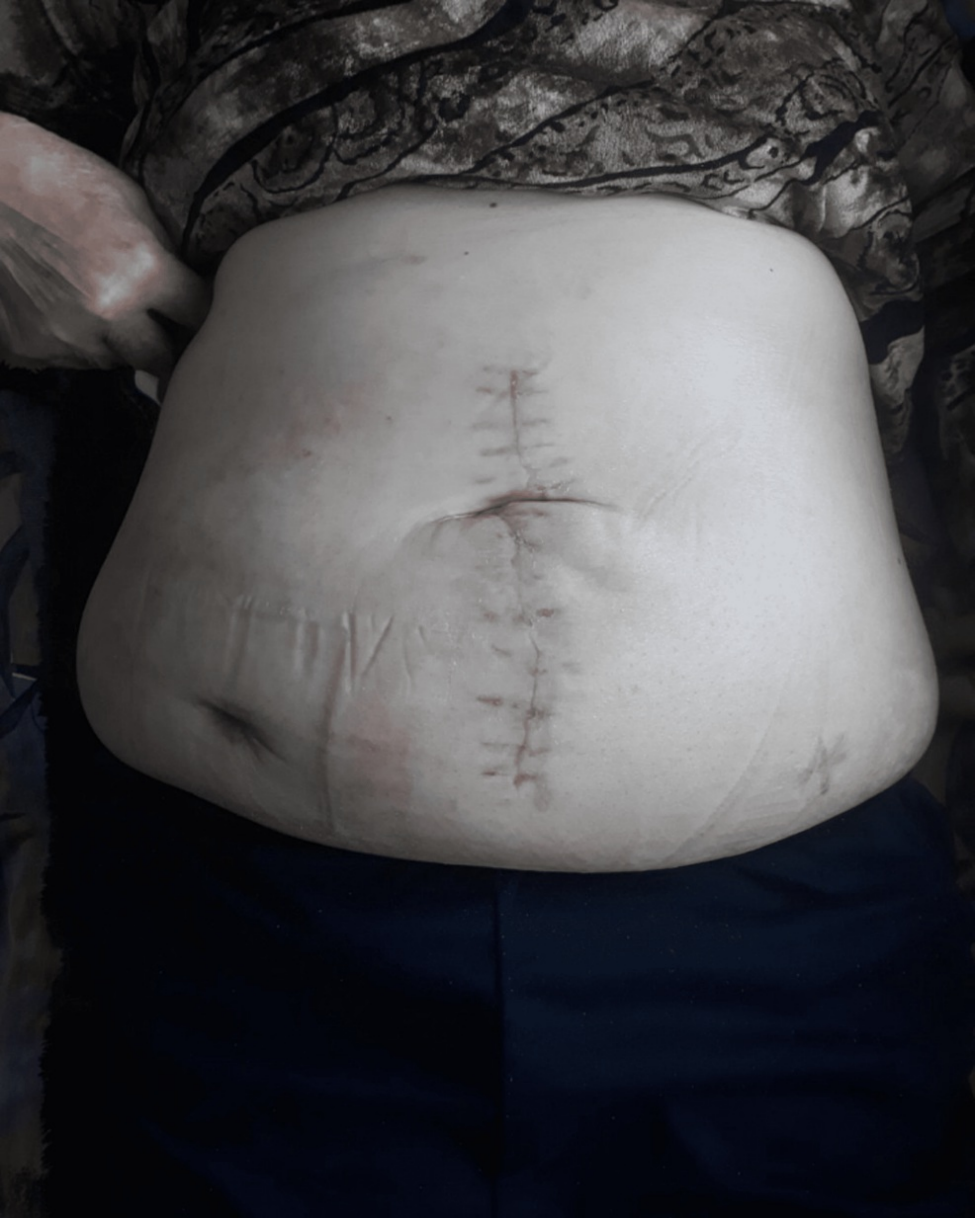
Two weeks after the reconstruction of the digestive continuity

One month later, a restoration of the continuity of the gastrointestinal tract with the creation of an L-L ileo-descendo anastomosis followed (Figure 8). The patient did well, the recovery was uneventful and she was discharged on the twelfth postoperative day. She decided against hernia repair due to the highly reduced risk of reincarceration and on account of her fear of operations (eventually required complex surgical repair). 

## Discussion

The first recorded mention of an umbilical hernia was made by Celsus, who in 100 AD called it “an indecent prominence of the navel” [8]. The umbilical hernia, also called exumbilication is a protrusion of abdominal content through the abdominal wall at the umbilicus. It should be distinguished from exomphalos or omphalocele, in which the intestines and organs protrude outside the belly and are covered only by a thin membrane (consisting of Wharton's jelly, peritoneum, and amnion). Patients with an umbilical hernia usually complain about pain in the umbilical region with a sudden onset of swelling. Incarceration of the omentum, from our experience, is often associated with a cough. Clinical diagnosis of strangulation is complicated because there could be a high discrepancy between the clinical presentation and the intraoperative findings, especially in older patients with, e.g., diabetes mellitus [6]. An early operation is important as a delay increases morbidity. Example gratia: A bowel perforation leads to consequential operative complications. It increases the length of the operation, the need for bowel resection, and the anastomosis creation and leads to prolonged recovery). [9]. It also limits the operative possibilities, meaning that operative field contamination reduces the possibility of mesh augmentation because of the possible mesh infection. If the mesh is to be used then it should be a low-weight polypropylene mesh. Umbilical hernias should be repaired using mesh, especially if a patient has multiple comorbidities that are significantly associated with recurrence, such as obesity, diabetes, liver disease, and ascites [10]. A higher incidence of SSI (surgical site infection) also leads to a consecutive higher hernia recurrence. Patients with intestinal strangulation have often associated peritonitis and there are critically ill cases, commonly shocked and at high risk of septic complications. These patients may experience high intraoperative intra-abdominal pressure. Such hypertension may be the underlying cause of reduced cardiac output, splanchnic hypoperfusion, and oliguria, leading to an abdominal compartment syndrome leading, visceral perfusion, and acute bowel injury [11-13]. An ileostomy creation is recommended in older, unfit patients with comorbidities and anastomosis creation is only when there is no sign of gangrene [11]. When early definitive fascial closure is not possible, progressive closure can be gradually attempted at every surgical wound revision. A skin-only closure is a viable option and subsequent eventration can be managed at a later stage with delayed abdominal closure and synthetic mesh repair (grade 1C recommendation) [14]. 

## Conclusions

Giant hernias with large hernia necks can also incarcerate and strangulate. The presentation of strangulation, especially in older people, can occur with far fewer symptoms (pain) than expected. There is often a high discrepancy between the clinical presentation and the intraoperative findings. This case highlights a unique dilemma, physicians managing chronic large abdominal hernias in the elderly are facing, which carries potentially high morbidity and mortality with minor symptoms and subtle clinical findings. As the population of the elderly is growing worldwide, further research is urgently needed to establish criteria to clearly recognize such cases. An urgent operation should always be undertaken in case of incarceration. Always thinking of possible strangulation, independent of the clinical unreliability of the presentation (absence of abdominal tenderness) and the anamnestic unreliability of the data.
